# Raman Spectroscopy of Optically Trapped Single Biological Micro-Particles

**DOI:** 10.3390/s150819021

**Published:** 2015-08-04

**Authors:** Brandon Redding, Mark J. Schwab, Yong-le Pan

**Affiliations:** U.S. Army Research Laboratory, 2800 Powder Mill Road, Adelphi, MD 20783, USA; E-Mails: redding.brandon@gmail.com (B.R.); mark.schwab@yale.edu (M.J.S.)

**Keywords:** optical trapping, Raman spectroscopy, bioaerosols

## Abstract

The combination of optical trapping with Raman spectroscopy provides a powerful method for the study, characterization, and identification of biological micro-particles. In essence, optical trapping helps to overcome the limitation imposed by the relative inefficiency of the Raman scattering process. This allows Raman spectroscopy to be applied to individual biological particles in air and in liquid, providing the potential for particle identification with high specificity, longitudinal studies of changes in particle composition, and characterization of the heterogeneity of individual particles in a population. In this review, we introduce the techniques used to integrate Raman spectroscopy with optical trapping in order to study individual biological particles in liquid and air. We then provide an overview of some of the most promising applications of this technique, highlighting the unique types of measurements enabled by the combination of Raman spectroscopy with optical trapping. Finally, we present a brief discussion of future research directions in the field.

## 1. Introduction

Raman spectroscopy relies on measuring the frequency and relative intensity of inelastically scattered light due to the vibrational, rotational, and other low-frequency modes of a sample. As such, the Raman spectrum provides a fingerprint of the molecules present in a sample [1,2]. Raman spectroscopy has been broadly used as one of the main diagnostic techniques in analytical chemistry and is developing into an important method in biology and medicine as a real-time clinical diagnostic tool for the identification of disease, and evaluation of living cells and tissue [1]. In addition, Raman spectroscopy is a promising method for the identification of aerosolized biological and chemical threat agents. 

The primary challenge associated with performing Raman spectroscopy is the inefficiency of the Raman scattering process, which results in a signal ~100 dB weaker than typical fluorescence [2]. Hence, spontaneous Raman measurements require a long signal integration time and can be difficult to perform on individual cells or particles in a solution or in the air which do not remain in the same position long enough to acquire a Raman spectrum. One solution to this challenge is to deposit the particle or cell of interest on a substrate before the measurement [3]. However, this has clear limitations since the substrate can alter the Raman spectrum of the particle, limit the ability to perform longitudinal studies of a particle in its natural environment, or introduce a background Raman signal, making it difficult to isolate the Raman spectrum from the particle of interest [4]. Moreover, dense particle deposition introduces challenges when trying to obtain the Raman spectrum from a single particle.

The combination of laser trapping with Raman spectroscopy (LTRS) circumvents these issues by holding a particle or cell in place long enough for data acquisition. Since optical trapping is possible in both solution and air, the potential influence of inelastic scattering from the substrate is avoided [4]. Since the Raman spectrum of a trapped particle can be measured *in situ*, studies on the temporal response of a particle to environmental changes are possible [5]. In addition, particle trapping using laser tweezers holds the particle near the high intensity portion of the beam, simplifying the alignment by maximizing the Raman signal. Such a combined method also enables the study of individual particles, providing information about the heterogeneity of a population which can be difficult to extract from a Raman measurement of a bulk sample [6]. Performing LTRS on relatively large biological particles can even enable the measurement of the molecular content of different regions of a cell [7].

While LTRS has been performed on a wide range of particle types, in this review we will focus on its application to the characterization of biological particles. Biological aerosol particles, or bioaerosols, have important implications for human health, acting as airborne disease transmitters that contain microorganisms such as bacteria, viruses, pollen, and fungi. Monitoring bioaerosols in locations such as hospitals for the presence of airborne diseases, or public spaces for the detection of aerosolized biological warfare agents are increasingly important problems. Aerosol particles also have significant implications for climate change due to their role in the scattering and absorption of solar radiation as well as in cloud condensation and the formation of ice nuclei. Thorough characterization of the composition and density of aerosol particles is therefore essential to the accuracy of climate change models. Raman spectroscopy, particularly when combined with optical trapping, is uniquely suited to the characterization of bioaerosols due to its combination of high specificity with a modest cost and non-invasive nature. 

LTRS is also emerging as a powerful tool in molecular biology due to its ability to perform longitudinal studies on individual cells, spores, bacteria, and viruses in their natural environments. Bioaerosols are a complex mixture containing numerous biomolecules in various concentrations and forms. Previous single-particle optical characterizations using fluorescence were only able to probe a limited range of biological compounds, including proteins, amino acids (tyrosine, tryptophan *etc*.), nucleic acids (DNA, RNA *etc*.), coenzymes (nicotinamide adenine dinucleotides, flavins, and vitamins B_6_ and K and variants of these), polysaccharides, dipicolinates, and lipids. Raman spectroscopy, especially when long acquisition times are enabled through LTRS, can characterize a much broader range of biomolecules and with higher specificity compared to techniques that probe only fluorescent compounds. Some cells or spores can grow, change, and reproduce in buffer liquid or in air, and LTRS enables the study of these cells as they undergo these processes. For example, as a cell grows, some biomolecules can decrease or vanish, while others increase or can even be generated. Therefore, using Raman spectroscopy to detect and monitor specific biomolecules within a cell as it responds to changes in its environment can provide new insights into our fundamental understanding of cell growth. LTRS is also emerging as an important tool in drug discovery due to its ability to monitor a cells response, for example, to varying forms of chemotherapy [8]. 

In this paper, we provide a brief review of techniques that perform Raman spectroscopy on individual optically trapped biological particles. We discuss many of the promising applications of LTRS and attempt to highlight the unique features of LTRS which make it such a powerful technique. This paper is organized as follows: in Section 2, we present a discussion of the most common optical trapping techniques used in LTRS; in Section 3 we discuss the development of LTRS as well as several exemplary applications. In Section 4, we provide a brief overview and discussion of future applications and research directions. We hope this review will provide researchers entering the field with an introduction to the wide array of LTRS applications as well as its key features. 

## 2. Optical Trapping Techniques

**Figure 1:**
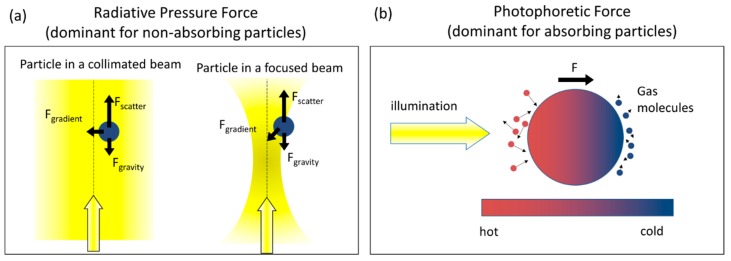
(**a**) The radiative pressure force, which is the dominant force experienced by non-absorbing particles, results from the transfer of momentum from photons scattered by a particle. The radiative pressure force can be divided into a scattering force, which tends to push the particle along the direction of light propagation, and a gradient force, which tends to pull the particle toward the highest intensity region. The gradient force enables trapping in a focused laser beam; (**b**) The photophoretic force, which is the dominant force experienced by strongly absorbing particles, results from the transfer of heat to surrounding gas molecules from a non-uniformly heated and/or non-uniformly heat-emitting particle.

Optical tweezers rely on the radiative pressure force which results from the transfer of momentum from photons to a particle. In Figure 1a, we illustrate the influence of the radiative pressure force on a particle in a collimated beam and in a focused beam. The radiative pressure force is often divided into a scattering force and a gradient force, although both result from the same transfer of momentum from the incident photons [9,10]. The scattering force tends to push particles in the direction of light propagation whereas the gradient force tends to pull the particle towards the high intensity region. Ashkin’s original demonstration relied on the radiative pressure acting on “relatively transparent particles in a relatively transparent media” to avoid thermal effects which “are usually orders of magnitude larger than radiation pressure” for strongly absorbing particles [11]. Absorbing particles are subject to a photophoretic force which results when an absorbing particle is non-uniformly heated and/or non-uniformly heat-emitting. As illustrated in Figure 1b, a strongly absorbing particle is non-uniformly heated if it is illuminated from one side. When the heat is transferred to the surrounding gas molecules, gas molecules on the warmer side of the particle will acquire more energy and subsequently collide with the particle at higher velocity, imposing a net force pushing the particle toward its cold side. This photophoretic force can be 4–5 orders of magnitude stronger than the radiative pressure force [12] and is therefore the dominant force acting on strongly absorbing particles. 

### 2.1. Optical Trapping via the Radiative Pressure Force (Laser Tweezers)

In 1970, Ashkin first demonstrated that optical radiation pressure could be used to trap glass beads in water using two counter propagating, focused beams [11]. A year later, he demonstrated levitation of glass spheres in air and in vacuum using a vertically oriented beam to compensate gravity [13], and in 1976, Roosen *et al.* showed that the gradient force was strong enough to overcome gravity, enabling the trapping of solid glass spheres in two counter propagating horizontal beams [14]. The first single beam optical trap for an airborne particle (a 5 μm glass sphere) was demonstrated in 1997 by using an objective with a high numerical aperture (NA = 0.95) to provide a sufficiently strong gradient force [15]. Since these initial demonstrations the field of optical tweezers has experienced rapid growth and developed into an indispensable tool in the study and manipulation of micron sized particles [16].

**Figure 2:**
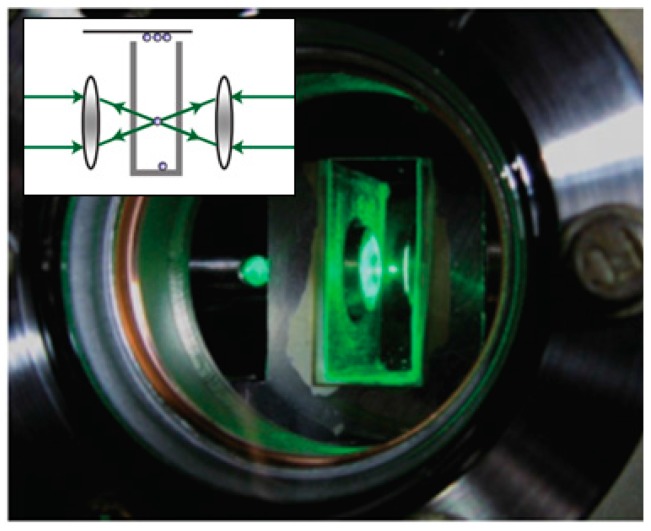
A 4.7 μm diameter microsphere trapped inside a vacuum chamber by a counter-propagating dual-beam optical tweezer. The wavelength of the trapping beams is 1064 nm; A weak green (532 nm) laser is used for illumination. Inset is a counter-propagating dual-beam optical trap in air based on radiative pressure forces. With kind permission from Springer Science and Business Media [9].

Radiative pressure based optical trapping techniques can be divided into single or multiple beam configurations. Single beam traps are more easily aligned; however, a high NA is typically required to enable optical trapping. This constraint is particularly pronounced when trapping particles in air, since the high refractive index contrast between the particle and air results in a strong scattering force which tends to destabilize the trap [9,17]. Using two counter-propagating beams to cancel out the scattering force enables optical trapping of airborne particles with much lower NA (Figure 2); however the alignment in such systems can be very critical [9]. 

Radiative pressure traps have been demonstrated with both continuous wave (CW) and pulsed lasers. Although the average power was found to be the primary factor dictating the efficacy of the optical trap [18], trapping using a pulsed laser may have advantages in potential non-linear optical applications.

### 2.2. Optical Trapping via the Photophoretic Force

**Figure 3:**
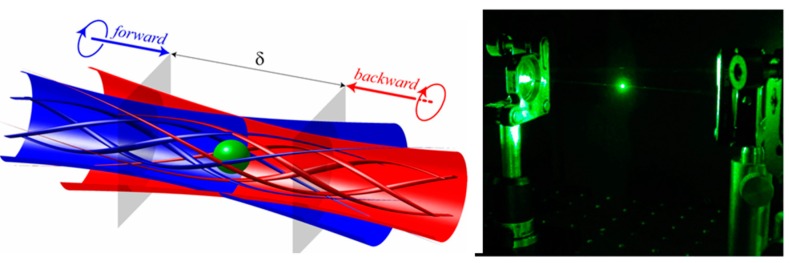
An example of a photophoretic trap. The particle is trapped in the low intensity region between two counter-propagating Laguerre-Gaussian vortex beams [21] (Fair Use according to OSA).

The photophoretic force can provide a highly stable optical trap even for airborne particles. Optical levitation based on the photophoretic force was demonstrated as early as 1982 [12] and photophoretic trapping in a low-light optical vortex in 1996 [19]. In recent years, a number of additional techniques have been developed which utilize the photophoretic force to trap airborne particles. Unlike laser tweezers, optical traps based on the photophoretic force generally trap absorbing particles in a low-light intensity region where the particle is surrounded by light in 3-dimensions, as in the example shown in Figure 3 where a particle is trapped between two counter-propagating vortex beams [20,21,22]. Additional methods to generate such a low-light intensity region include hollow cones formed by a ring illuminating the back aperture of a lens [23,24], a low-light region formed between two counter-propagating hollow beam [24], tapered rings [25], optical lattices [26], bottle beams [27], and even speckle fields [28]. Although absorbing particles were trapped in the low-light region in each of these demonstrations, there have also been a few recent demonstrations of optical trapping in the high-intensity portion of a single focused beam [29,30]. To explain the origin of this phenomena, researchers have cited the role of the accommodation coefficient, which describes the ability of a particle to transfer heat to the surrounding gas molecules [31,32,33]. The accommodation coefficient depends on the material and morphology of a particle. If the accommodation coefficient varies along the surface of a particle, a body-centric force can result even in a uniformly heated particle. Moreover, the accommodation force can at times be orders of magnitude stronger than the “longitudinal” photophoretic force (*i.e*., the force shown in Figure 1b) [32], and could explain anomalous observations such as a “negative” photophoretic force experienced by strongly absorbing particles [34,35]. 

### 2.3. Alternate Trapping Modalities

Holographic optical tweezers enables many particles to be trapped and manipulated simultaneously. It was first demonstrated using a fixed diffractive optical element [36,37,38]. However, the functionality of holographic optical tweezers was greatly enhanced by the development of spatial light modulators which enabled researchers to rapidly update the optical trapping pattern without the need to fabricate a new diffractive optical element [38,39]. This approach was also applied to trap aerosol droplets [40].

**Figure 4:**
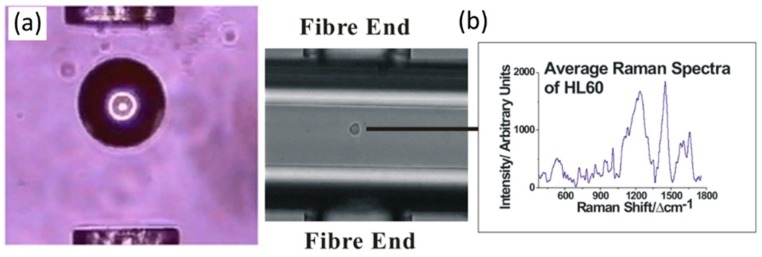
(**a**) A 100 µm polymer sphere is trapped between two fibers; (**b**) A HL60 cell is trapped in a microfluidic channel between two fibers. The particle is stopped in flow while the Raman spectrum is recorded and then released [41] (Fair Use according to OSA).

Recently, optical fibers have also been proposed as a mechanism to achieve optical trapping. For example, Jess *et al.* [41] showed that a particle could be trapped in the diverging beams between two multimode fibers directed toward each other, as shown in Figure 4. This method enabled the manipulation of larger cells (up to 100 μm in diameter) than can be trapped in most optical tweezers systems [41]. A separate microscope objective was then used to collect the Raman spectra of the trapped particles, providing a means to collect Raman spectra from different positions within a trapped cell. Analysis of the spatially varying Raman spectra within the cell were used to allow for the identification of the nucleus, cytoplasm, and membrane regions of the cell using a principal component analysis (PCA) [41]. The dual fiber trap was also extended to trap and record the Raman spectra from particles in microfludic flow channels, as shown in Figure 4. This illustrates the potential for LTRS to be used for the on-line characterization of particles in a microfluidic system.

Optical trapping has also been demonstrated using an individual multimode fiber. In this configuration a spatial light modulator controlled the wavefront of light coupled into the fiber to form a focal spot (or several focal spots) at the distal end of the fiber to enable optical trapping. In this way, the system resembled holographic optical tweezers extended through a multimode fiber [42,43]. 

### 2.4. Trapping both Transparent and Absorbing Particles in Air Using a Single Shaped Laser Beam 

**Figure 5:**
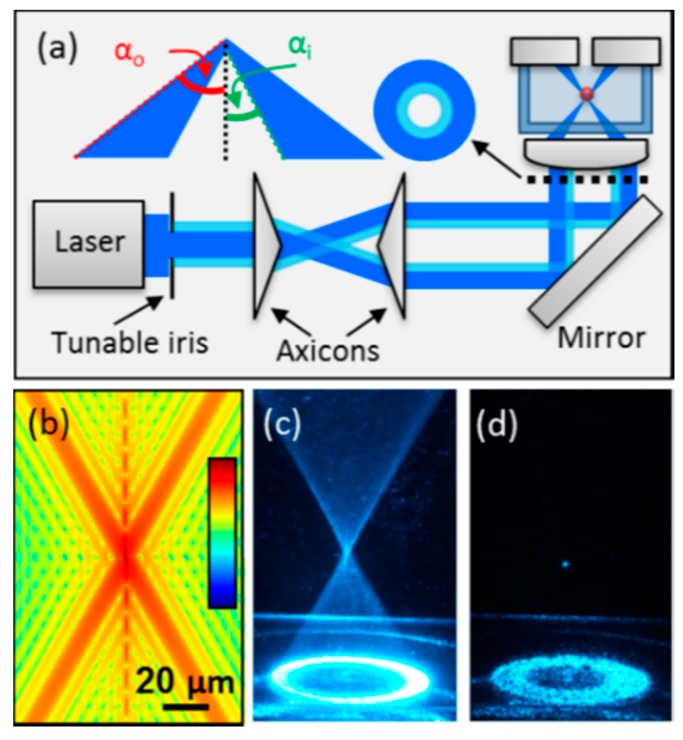
(**a**) Schematic of the optical trapping apparatus used to trap both transparent and absorbing airborne particles of arbitrary morphology using a single shaped hollow laser beam. The aspheric lens forms a hollow conical focus within a glass chamber where airborne particles are trapped; (**b**) Calculated intensity profile near the focal spot plotted on a log-scale; (**c**) Image of the conical focal region produced inside the chamber obtained by introducing Johnson Smut Grass Spores and recording a long exposure time image; (**d**) Image of a spore trapped in air near the focal point [44] (Fair Use according to OSA).

Due to the distinct nature of the radiative pressure and photophoretic forces, most optical traps formed by a single laser beam are designed for either trapping absorbing or transparent particles. However, many applications require the ability to trap particles regardless of their morphology and absorptivity. Recently, a technique was shown to enable the trapping of both absorbing and transparent particles using a fixed optical geometry [44]. In this approach, a single shaped laser beam forms a hollow optical cone in which absorbing particles are trapped in the low-light-intensity region above the focal spot via the photophoretic force while non-absorbing particles are trapped at the high-intensity focal spot via the radiative pressure force. The experimental trapping apparatus used to realize this optical trap is shown in Figure 5a along with the calculated intensity profile near the focal spot (shown on a log-scale), an image of the conical focal region, and an image of a Johnson smut grass spore trapped in air [44]. This approach also reduces the scattering force near the focal spot, thereby enabling radiative pressure based trapping of transparent particles with lower NA (e.g., N ~ 0.55 for a particle with refractive index of 1.5) compared with traditional laser tweezers which require NA ~ 0.9 [17,44,45]. This approach was first used to trap droplets in air [45] and later shown to trap solid, transparent particles such as glass beads and albumin in air, as well as absorptive particles such as fungal spores [44]. Moreover, since particles of each type are trapped along the optical axis, this method could be combined with a particle interrogation technique such as Raman spectroscopy by imaging the optical axis to the entrance slit of a spectrometer. The ability to trap airborne particles in a fixed optical geometry regardless of the particle morphology or absorptivity could enable extensive on-line characterization of bioaerosols. 

In addition to the optical techniques discussed above, bioaerosols particles can also be trapped using magnetic [46], electrodynamic [47], and acoustic forces [48]. However, in this article, we will limit our discussion to optical trapping techniques and their integration with Raman spectroscopy. 

## 3. Laser Trapping Raman Spectroscopy (LTRS) 

### 3.1. Development of LTRS

**Figure 6:**
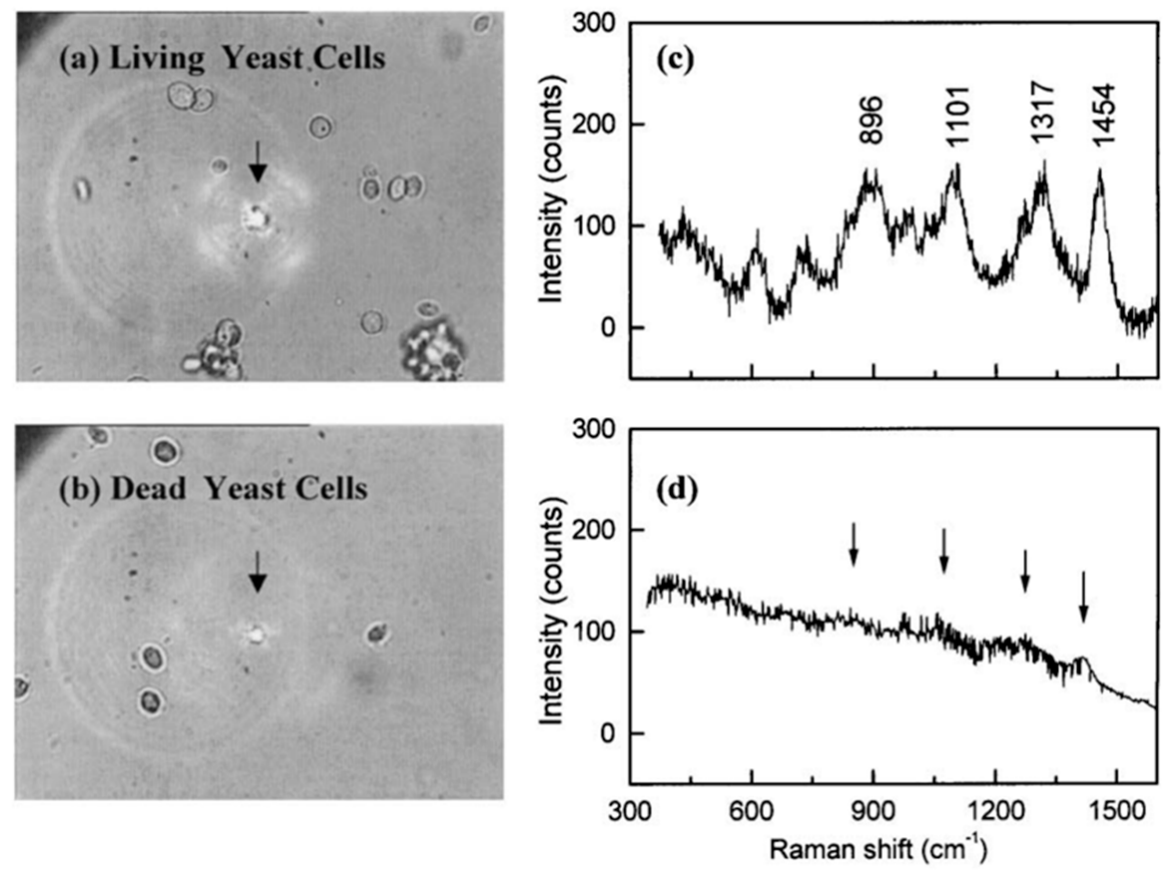
Raman spectra of trapped yeast cells revealing distinct spectra depending on whether the yeast cells are alive or dead [54] (Fair Use according to OSA).

Raman spectroscopy was first combined with optical trapping in a 1984 work in which the Raman spectra were measured from levitated glass spheres and quartz microcystals in air [4]. Soon after, optical trapping was used to obtain information about the molecular structure from single microdroplets [49,50]. Later, a near-infrared (NIR) laser source was shown to reduce the fluorescence background and photo-damage effects on live cells, although it increased the alignment complexity and the instrument cost [51,52]. The first study performed on biological particles was not conducted until 2002 when LTRS was demonstrated on single cellular organelles [53] as well as on living blood cells and yeast cells [54]. Soon afterwards, it was applied to obtain surface-enhanced Raman scattering (SERS) from single optically trapped bacterial spores [55]. As an example, Figure 6 shows the Raman spectra recorded from optically trapped yeast cells, illustrating the ability of Raman spectroscopy to differentiate between live and dead yeast cells which are essentially indistinguishable from the microscope image. 

A 2003 study demonstrated the ability of LTRS to study the behavior of a single cell over time as it responded to environmental changes [5]. In particular, the response of single cells of *Escerichia coli* and *Enterobacter aerogenes* bacteria to changes in temperature was studied. The study observed significant changes in the phenylalanine band which was attributed to heat denaturation of proteins [5]. The temporal dynamics of yeast cells exposed to changing temperatures were studied via LTRS a year later [56]. Raman spectra of the trapped yeast cells showed irreversible changes in two of the Raman lines (1004 and 1604 cm^−1^) as temperature increased from 25 °C to 80 °C [56]. 

**Figure 7:**
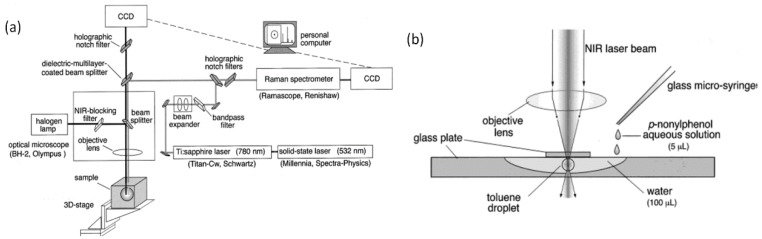
One of the earliest typical LTRS experimental schematics for (**a**) the near-infrared Raman trapping system; and (**b**) the optical arrangement for the sample cell [52] (With permission from ACS publications).

Although there are various optical arrangements used for LTRS, most of them are composed of a few key components, as exemplified in one of the earliest LTRS systems shown in Figure 7 [52]. The LTRS system consists of a laser source for trapping and potentially a second laser source for Raman excitation; a microscope to focus the trapping laser beam, image the trapped particle, and collect the Raman signal; a spectrograph/spectrometer or monochromator; and a photoelectronic detector (charge-coupled device (CCD), Image-intensified CCD (ICCD), electron multiplying CCD (EMCCD), photomultiplier tube (PMT), or avalanche photodiode (APD)) to record the Raman spectra. In order to minimize the elastic scattering from the trapping and exciting laser while maximizing the Raman signal, a notch filter, long-pass filter, or dichromatic filter is usually required. Since laser tweezers traps particles near the focal point of the objective lens, the particles are necessarily aligned in the high intensity part of the beam, enabling efficient Raman excitation. As a result, most LTRS systems use the trapping laser to also act as the Raman excitation light source [51,52,57] although a second laser can provide additional flexibility [49,53]. Although photophoretic trapping tends to confine the particle in a low intensity region, Raman spectra have nonetheless been measured with a few second integration time using a single beam to provide both the photophoretic trap and Raman excitation [58]. 

However, some trapping methods have relatively large fluctuations in the particle trapping position (e.g., over a few 10 s of µm), which could reduce the amount of Raman scattered light imaged onto the entrance slit of the spectrometer, making a Raman measurement impractical even with very long integration times (e.g., ~10 s). To overcome such a problem, researchers have introduced a position sensitive detector to monitor the particle position and provide feedback to adjust the laser power in order to hold the particle in a fixed trapping location [58]. Other LTRS systems take advantage of advanced microscopy techniques such as confocal, differential interference contrast, and phase contrast to provide additional functionality or improve the signal to noise ratio of the Raman spectra. For example, combining LTRS with a confocal microscope can efficiently reject out of focus light to improve the Raman signal [3,57,59,60]. Recording phase contrast images in addition to the Raman spectra has provided additional information about the refractility of spores [61,62].

**Figure 8:**
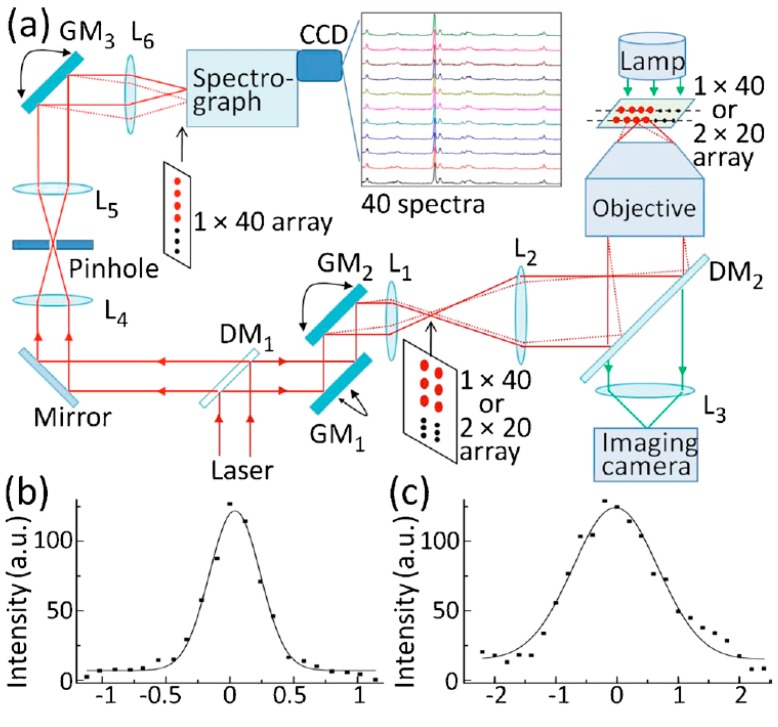
(**a**) Schematic of a multifoci-scan confocal Raman imaging system; (**b**) Lateral; and (**c**) axial intensity profiles of the Raman band at 1001 cm^−1^ of a 100 nm diameter polystyrene bead [57] (With permission from AIP Publishing LLC).

Since multiple particles can be trapped and manipulated simultaneously (e.g., by a holographic or diffractive optical pattern), the combination with LTRS enables longitudinal studies of the interaction between multiple bioaerosols. It also enables studies of the heterogeneous response of different individual particles to a stimuli [57,63,64]. Figure 8a presents a typical schematic of a multifoci-scan confocal Raman imaging system, which relies on a set of galvo-mirrors to rapidly steer the beam in order to generate 40 focal spots under the microscope, each of which is used to trap an individual bacteria spore. The Raman spectra of each of these spores can then be recorded in parallel on an imaging spectrometer by using a third galvo to direct the Raman signal from different particles to different positions along the entrance slit of the spectrometer [57]. 

### 3.2. LTRS Studies on Blood Cells

Red blood cells have been frequently studied by LTRS. An early study on the effects of photodamage on trapped red blood cells found that a blood cell trapped with ~2 mW continued to show the same characteristic Raman spectrum for 30 min, whereas a cell trapped with ~20 mW showed a dramatic change in the Raman spectrum after ~15 min, indicating the onset of photodamage [54]. 

**Figure 9:**
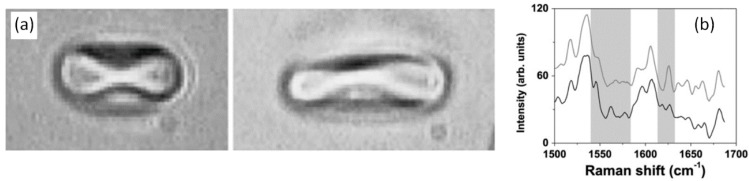
(**a**) A red blood cell is stretched using optical tweezers while the Raman spectra is monitored to gauge the cell oxygenation level; (**b**) The Raman spectra of the stretched (bottom curve) and un-stretched (top curve) blood cell. The shaded regions highlight Raman bands which were most affected by mechanical stretching [65] (With permission from Elsevier).

LTRS was used to study the effect of mechanical strain on oxygenation in red blood cells [65]. In this study, the optical tweezers were used to apply a force, stretching a red blood cell, while the Raman spectrum was used to provide a measure of the oxygenation in the cell. The cell was stretched using two optical traps while a third beam was used to provide Raman excitation. Microscope images of the trapped cell before and after mechanical stretching are shown in Figure 9. This study was the first direct measurement of the relationship between optical force and oxygenation, highlighting the unique capabilities of an LTRS system to hold and manipulate a biological particle while simultaneously characterizing its chemical composition.

LTRS has also been used to study blood diseases such as thalassemia [66]. The LTRS study confirmed predictions of reduced oxygenation in thalassemic blood cells and identified differences in the Raman spectra of normal and thalassemic blood cells. In addition, the optical trapping apparatus was used to stretch the blood cells in order to measure the mechanical properties, confirming predictions of increased rigidity in thalassemic blood cells. This highlights the ability of LTRS to study both the chemical and mechanical properties of biological particles in a single experimental setup. LTRS also has potential as a diagnostic tool, and has been shown to differentiate between normal and malaria infected red blood cells [67].

**Figure 10:**
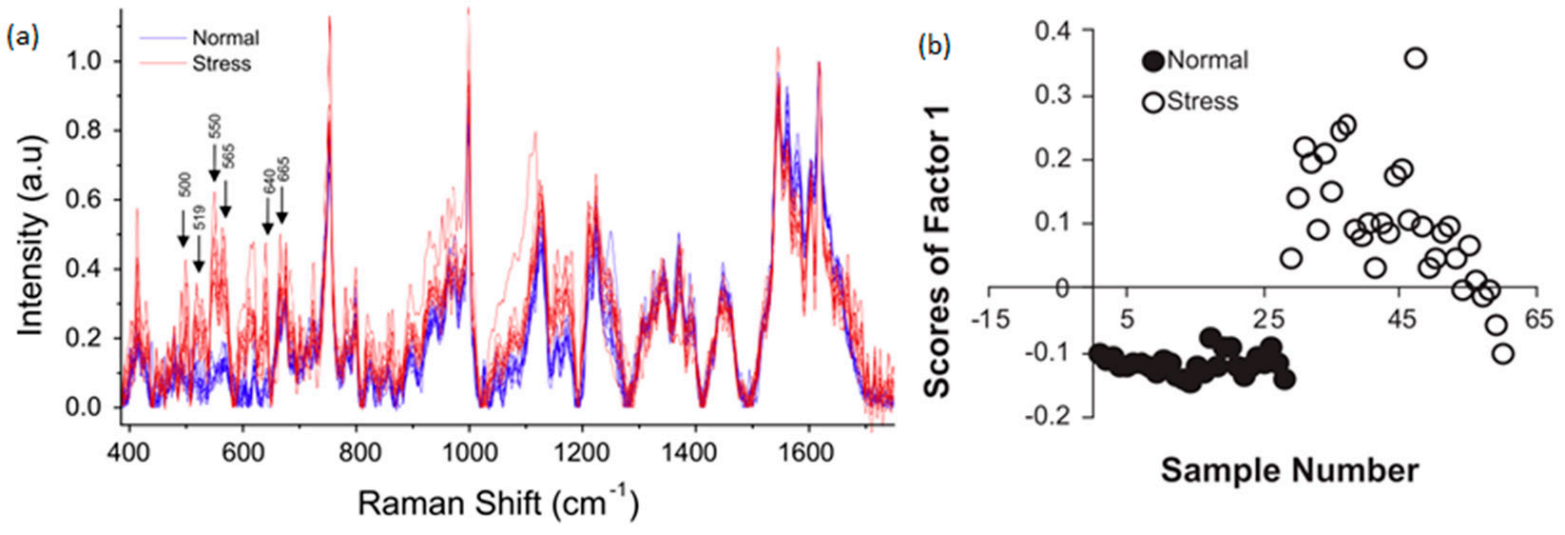
(**a**) Raman spectra from 10 normal cells and 10 cells exposed to oxidative stress. The variations between each spectra illustrate the cell-to-cell variation. Nonetheless, despite broadly similar Raman spectra; a PCA shown in (**b**) clearly differentiates between the stressed and unstressed cells [68] (With permission from Elsevier).

The effects of oxidative stress were studied on red blood cells using LTRS by Zachariah *et al.* [68]. The Raman spectra of 10 normal blood cells and 10 cells exposed to oxidative stress were recorded. Although the Raman spectra exhibited significant cell to cell variation, a PCA of the spectra enabled discrimination between the normal and stressed cells, as shown in Figure 10.

**Figure 11:**
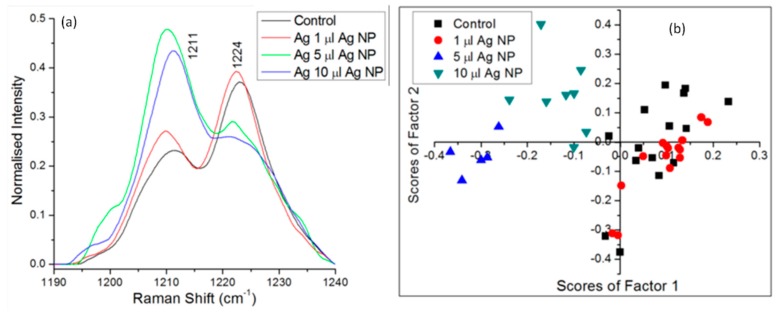
(**a**) LTRS analysis of red blood cells exposed to Ag nanoparticles revealed a change in the relative intensity of the 1211 and 1224 cm^−1^ lines, indicating a change in the methane C-H deformation region of the cell; (**b**) A PCA provided further insight into the temporal evolution of blood cells exposed to Ag nanoparticles [69] (Reprinted under the Creative Commons Attribution License).

A 2014 study investigated the effect of Ag nanoparticles on red blood cells [69]. Ag nanoparticles have potential anti-microbial applications and are also frequently used in producing surface-enhanced Raman scattering, but their effect in a biological context is not fully understood. Using LTRS, the Raman spectra of trapped red blood cells were measured after exposure to varying concentrations of Ag nanoparticles. As shown in Figure 11, sufficiently high concentrations of Ag nanoparticles altered the relative intensity of the Raman lines at 1211 and 1224 cm^−1^, corresponding to a change in the methine C-H deformation region of the cell (among other changes) [69]. Monitoring the Raman spectra of exposed blood cells showed that the Ag nanoparticles produce irreversible changes through a transformation from an oxygenated to a de-oxygenated state. By providing information about the temporal evolution of the chemical structure of the blood cells, LTRS provided insights into the cell/nanoparticle interaction process [69].

LTRS has also been performed *in vivo* on red blood cells within the microvessel of a mouse ear [6]. This enabled researchers to measure the relative oxygenation and pH of blood cells in the arterioles and venules. They also compared the Raman spectra of cells measured *in vivo* with cells measured *in vitro* in physiological saline, identifying key differences and highlighting the importance of *in vivo* studies. In addition LTRS enabled a non-destructive measurement without requiring blood extraction [6]. 

### 3.3. LTRS Studies of Yeast Cells

**Figure 12:**
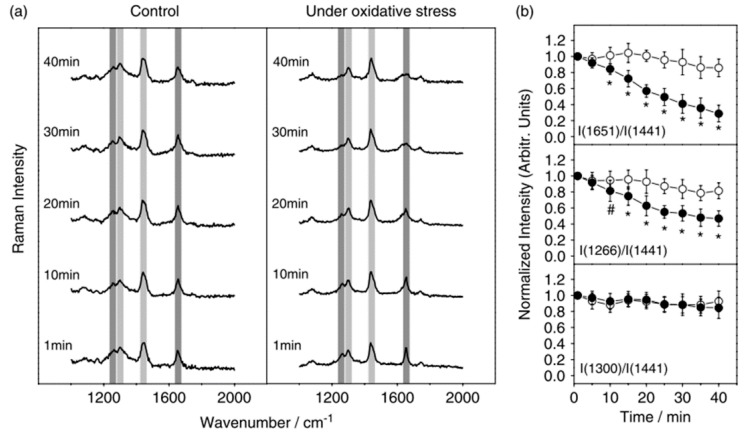
(**a**) The temporal response of yeast cells to oxidative stress is characterized via LTRS; (**b**) Raman lines associated with varying chemical bonds within the yeast cell are monitored over time. While the bonds associated with the Raman line at 1651 cm^−1^ and 1441 cm^−1^ are diminished, the bonds associated with the line at 1300 cm^−1^ (among others) are unaffected [70] (With permission of John Wiley & Sons).

Yeast cells have also been investigated by several groups using LTRS since a 2002 study revealed differences in the Raman spectra of live and dead trapped yeast cells [54]. Later, a detailed study showed the response of a trapped yeast cell (*Pichia pastoris*) to oxidative stress over time [70]. This result indicated that Raman lines (e.g., 1651 cm^−1^ and 1266 cm^−1^) associated with C=C stretching and =CH deformation are reduced under exposure to oxidative stress, whereas lines associated with the twisting and bending modes of CH_2_ remained relatively unaffected. The temporal dependence of varying Raman lines to oxidative stress are shown in Figure 12. The ability of ascorbic acid to mitigate the effects of oxidative stress was also investigated, illustrating the potential of LTRS to evaluate potential therapeutics [70]. 

### 3.4. LTRS Studies on Biological and Bacterial Spores

**Figure 13:**
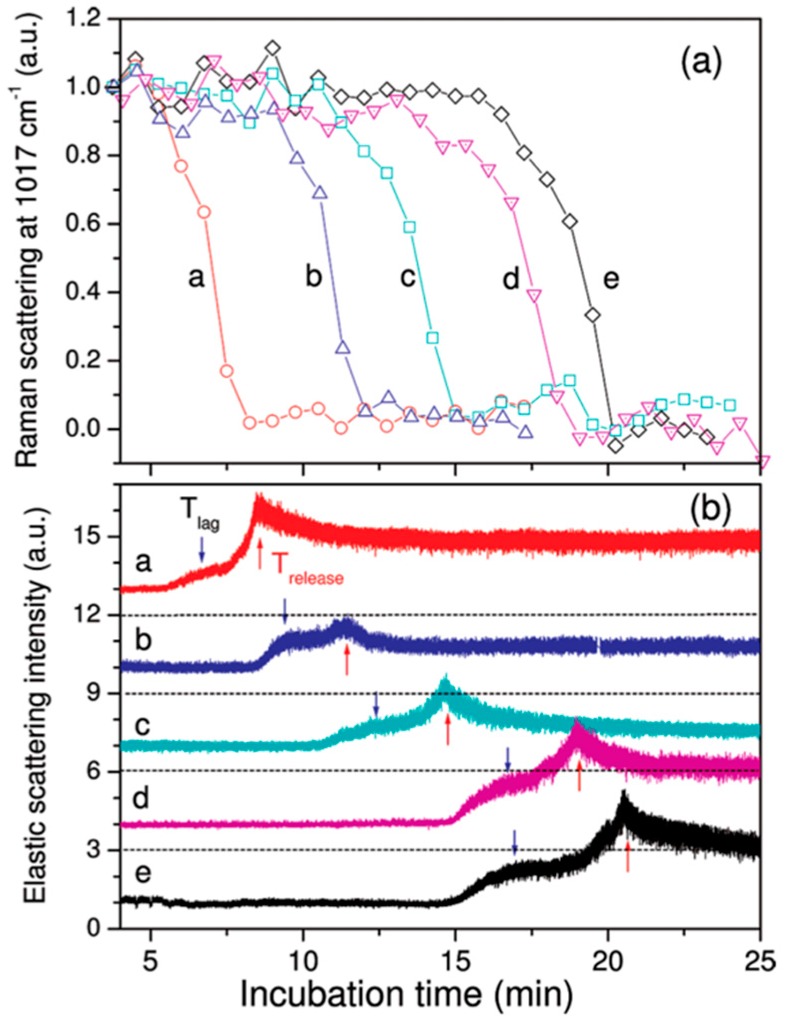
The Raman line (**a**) associated with the calcium dipicolinate biomarker is monitored during the spore germination process along with the intensity of elastic scattered light; (**b**) for varying particles. The individual particles show different germination times, indicated by the rapid decrease in the Raman scattering line at 1017 cm^−1^ (**a**); but the germination process is consistently correlated with an increase in the elastic scattering of the cell (**b**) [61]. (With permission from ACS publications).

LTRS has been used to study the germination process in *Bacillus* spores by monitoring the time varying calcium dipicolinate biomarker in the Raman spectra [71]. Monitoring numerous individual cells provided information about the variation in the time to germination of individual spores. In addition, the studies of individual cells revealed that the calcium dipicolinate biomarkers were rapidly released in individual spores, albeit at different times for different spores, whereas Raman measurements averaged over a population of spores showed only a smooth decay in the presence of the biomarker [71]. A later study by the same group combined LTRS with measurements of the elastic scattering properties of a *Bacillus* spore during germination to provide additional information about changes in the morphology and refractive index of the spore [61]. As shown in Figure 13, they were able to correlate changes in the elastic scattering of a spore with internal chemical changes monitored via the Raman spectra. 

A later study combined LTRS with phase contrast microscopy, providing the first clear demonstration of the correlation between the release of calcium dipicolinate and a change in refractility from bright to dark in the phase contrast images. They found that 70% of the decrease in the intensity of the phase contrast image coincided with the decrease in the calcium dipicolinate Raman line [62]. Additional studies have been performed on the development of *Geobacillus stearothermophilus* spores exposed to varying germinants [72]. LTRS has also been combined with measurements of changes in the speckle pattern formed by light scattered off a trapped cell [59] in a study which compared the dynamics of *E. coli* cells lysed from outside by an egg white lysozyme and from within by a temperature induced bacteriophage. The time varying Raman spectra revealed that the cell underwent significantly different responses in the cases considered. In addition, since the speckle pattern depends sensitively on the morphology of the cell, this provided additional information regarding the release of intracellular materials (e.g., proteins and ribosomes) which disrupted the cell wall.

**Figure 14:**
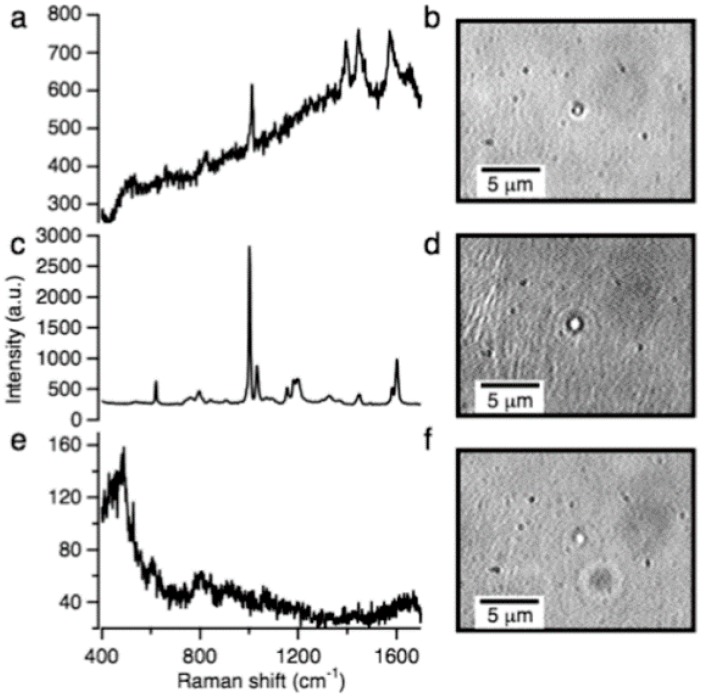
Raman spectra and microscope images of trapped particles of either (**a**,**b**) *Bacillus cereus* spores; (**c**,**d**) polystyrene microspheres; (**e**,**f**) glass microspheres. The LTRS system used the unique Raman spectra to rapidly identify the particle type [73] (With permission from ACS publications).

LTRS has also been used for the identification of bacterial spores in an aqueous environment with a mixture of additional particles [73]. Specifically, the LTRS system was able to identify *Bacillus cereus* spores in a mixed solution of similarly sized polystyrene and silica particles, despite indistinguishable microscope images, as shown in Figure 14. The LTRS-based identification system was validated by sampling 100 particles and found to correctly identify the fraction of each particle type in the mixture. This demonstrated the potential for such an LTRS system as a particle analyzer, possibly in a flow cytometry environment [73]. The LTRS system has the potential for much higher speed particle identification than methods based on cell cultures, and far superior specificity compared with fluorescence based particle identification schemes.

### 3.5. LTRS Used for Drug Discovery and Evaluation

**Figure 15:**
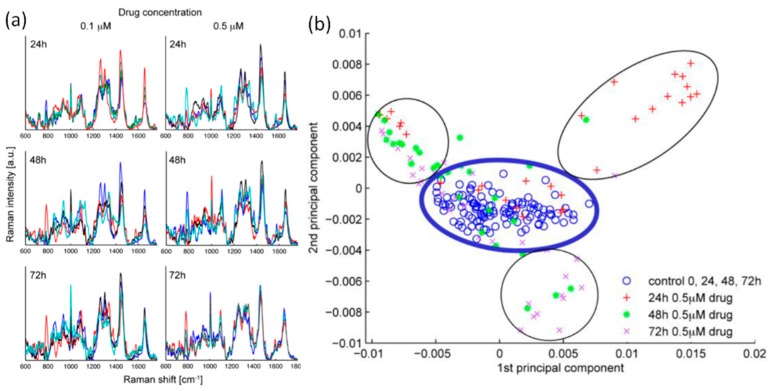
(**a**) Raman spectra recorded from leukemic T lymphocytes exposed to varying doses of a chemotherapy drug over time; (**b**) Principal component analysis revealed three stages in the apoptosis process induced by the drug [8] (Fair Use according to OSA).

LTRS also has tremendous potential as a tool in the evaluation and understanding of pharmaceuticals. A 2010 study used LTRS to evaluate the response of leukemic T lymphocytes exposed to the chemotherapy drug doxorubicin [8]. Raman spectra were recorded over 72 h after exposure to varying doses of the chemotherapy drug. Raman signatures indicative of changes in vesicle formation, cell membrane blebbing, chromatin condensation, and the cytoplasm of dead cells were observed during varying stages of apoptosis induced by the drug. Due to the heterogeneity in the cellular response, the individual Raman spectrum (shown in Figure 15) is difficult to interpret. However, a PCA was able to clarify the response of cells exposed to varying drug doses. This analysis revealed three distinct stages of apoptosis and the time required for the cell to progress through these stages depended on the drug dose. The ability of LTRS to study individual cells also revealed that certain cells did not respond to the drug and remained in the control group for the duration of the study, indicating that some cells either have a very slow response or exhibit a drug-resistant phenotype. This indicates a potential application of LTRS to rapidly determine if an individual patient will respond to a specific drug treatment. While this initial study recorded the Raman spectrum from a localized position within a cell [8], a follow-up study analyzed the Raman spectrum from the entire cell, further elucidating the cellular response to the drug [74].

In addition to characterizing the interaction between cancer cells and potential treatments, LTRS also has potential applications in the identification of cancer cells, as demonstrated in a study on using LTRS to identify epithelial cancer cells [75]. In this study, LTRS was performed on surgically removed human colorectal tissue revealing consistent differences in the Raman spectra of cancerous and non-cancerous cells through PCA. 

### 3.6. LTRS Studies on Airborne Bioaerosols

There is a high demand for real-time, in-situ detection and characterization of airborne bioaerosols based on single particle Raman spectroscopy. Raman spectroscopy has been shown to be able to discriminate between various microbes and bioaerosols; however, these measurements relied on first collecting the samples on a substrate and the Raman measurements were only performed after collection, limiting the characterization system response time and throughput [76,77,78,79]. LTRS has the potential to provide more efficient bioaerosol characterization, without the need for collection on a substrate, which could also interfere with the Raman spectra. However, optical trapping of a micro-particle in air is more challenging than in liquid because of the drag force in air and the larger optical scattering force due to the high refractive index contrast in air [9,24,44]. As a result, there have only been a few studies on airborne bioaerosol particles based on LTRS techniques, although there has been significant progress performing LTRS on airborne droplets which take advantage of the unique optical properties and morphology of airborne droplets. For example, several studies have investigated the effect of morphology-dependent microdroplet resonances on the Raman spectra, as well as studies on phase and size transitions, liquid-gas interactions, thermodynamic behavior, and the kinetics of mass transfer in airborne droplets [40,76,77,78,79,80,81,82,83,84,85,86].

**Figure 16:**
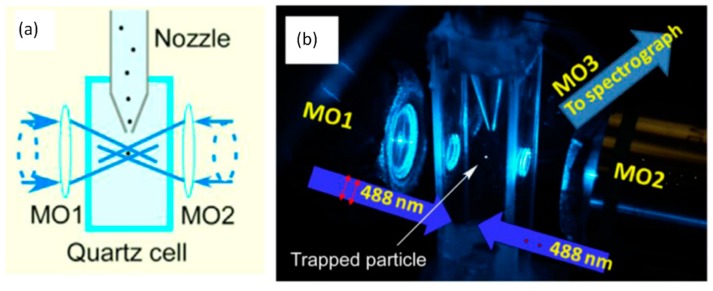
(**a**) LTRS studies on absorbing bioaerosols rely on a photophoretic trap to hold a particle in the low intensity region formed between two counter-propagating hollow beams; (**b**) Photograph of an experimental photophoretic LTRS system in which an aerosol delivery nozzle introduces particles into the photophoretic trap for on-line particle characterization [23]. (With permission from Elsevier).

Recently, photophoretic trapping was combined with Raman spectroscopy for the characterization and identification of absorbing bioaerosols, as shown in Figure 16. A 2012 study first presented this technique by measuring the Raman spectra of individual trapped carbon nanotube particles [58]. The Raman spectra of individual airborne carbon nanoclusters have also been measured in a single beam photophoretic trap [87]. A later study reported measurements of Raman spectra from individual bioaerosol particles (pollen particles and fungal spores) held in a photophoretic trap [23]. In these studies the trapping laser also provided the Raman excitation and the distinct Raman spectra could be used for particle discrimination and identification. The photophoretic trap was integrated with an aerosol delivery nozzle to enable efficient particle trapping for potential applications as an on-line aerosol characterization instrument [23,24]. Moreover, such photophoretic traps have been shown to work for a wide range of aerosol types, including biological molecules, proteins, fungal spores, and allergens [34].

LTRS has also been applied to clinical and medical research, further illustrating the potential impact of this technique [60,73,74,88,89]. The Raman spectrum is sufficiently sensitive to identify changes in an optically trapped stem cell due to the introduced stress of an attached nanoparticle [89]. In a work by Tong *et al.* [88], particles containing salbutamol, which is the active ingredient in inhalers used by asthmatics, were trapped in air within a high humidity environment to mimic the environment the particle experiences travelling from the inhaler to the lung. Raman spectroscopy was then applied to monitor the molecular changes in the particles as they interacted with the high humidity air while remaining optically trapped. Studies such as this enable a much richer understanding of the delivery of aerosolized pharmaceutical products. 

### 3.7. LTRS in Microfluidics and in Air for Continuously Sampling Bioaerosol Particles

The LTRS techniques we discussed above were primarily applied to study the physical, chemical, or biological properties from one, or a few representative bioaerosol particles, spores, or cells. These single particle studies relied on capturing and trapping individual particles from thousands of potential particles, either by passively waiting for a particle to enter the optical trap, or by actively selecting the particle. However, the ability to continuously trap, characterize, and release individual particles for on-line Raman-based particle identification or for longitudinal studies on a series of successively arriving particles with different properties could further increase the application space for LTRS, particularly if such a system could continuously sample individual particles from a particle stream (e.g., airborne particles from the atmosphere or particles in a liquid reservoir from a patient) over long periods of time [24]. 

There are two key requirements for such a system: (1) focusing and concentrating the particle stream into a small interrogation volume (e.g., 20 × 20 × 20 µm^3^) through which particles pass sufficiently slowly to be trapped and sampled one at a time; (2) the optical trap needs to be strong enough to capture and hold individual particles from the stream with different optical properties and morphologies. Toward these goals, a method was recently developed to deliver individual particles into the trapping volume based on counter-directional air flow which aerodynamically focused particles into the trapping position with minimal particle loss [24]. A second study showed that a single optical trapping technique could be used for both transparent and absorbing particles regardless of their morphologies [44]. Nevertheless, there has been significantly more success applying LTRS to particles in liquid, particularly in a microfluidic environment. These LTRS systems benefit from the confinement of the microchannel, which efficiently delivers particles to the trapping volume [41,90,91,92,93,94,95,96]. In addition to optical trapping, microfluidic systems have also been combined with electrostatic trapping [97,98,99]. Moreover, combining microfluidics with surface-enhanced Raman scattering (SERS) enables much faster Raman measurements, and it is possible to identify cells and characterize cellular chemical dynamics in flow, without needing to trap the particles [100,101,102,103,104,105]. 

**Figure 17:**
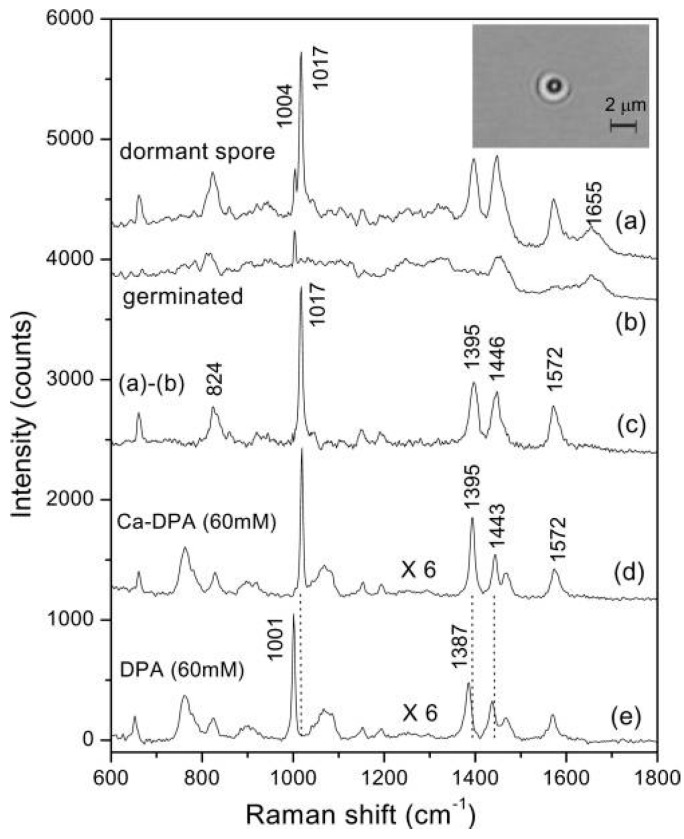
Raman spectra of (**a**) dormant; and (**b**) germinated *B. subtilis* spores; (**c**) subtraction of curve b from curve a; (**d**) The Ca-DPA; and (**e**) DPA Raman spectra [96] (With permission from American Society for Microbiology).

Continuous sampling systems based on LTRS in a microfluidic system tend to resemble the experimental apparatus shown in Figure 4, with the additional ability to store a reservoir of samples in a reservoir or syringe and gradually deliver these particles into the trapping volume. Such a system was shown to discriminate between cancerous and normal cells including erythrocytes, leukocytes, acute myeloid leukemia cells (OCI-AML3), and breast tumor cells BT-20 and MCF-7 [90,91]. In addition, the molecular compositions and structures of single cells or even the subcellular composition could be determined [41]. The physical and chemical mechanisms of many biological processes were also explored based on the interactions between cells as well as between cells and their environment [93]. A label-free cell sorting platform was developed based on intrinsic Raman markers for automated sampling and sorting of a large number of individual cells in solution [94]. Microfluidic based LTRS has been used to monitor the dependence of the neuronal action on nerve globins by obtaining the Raman spectra from several globin-containing cells: hemoglobin (Hb) within single red blood cells, a nerve globin present in the nerve cord of the annelid Aphrodite aculeata (A. aculeata), and wild-type (wt) human neuroglobin (NGB) overexpressed in Escherichia coli (*E. coli*) bacteria [95]. Calcium (Ca) ion-Dipicolinic acid (DPA) levels in individual trapped *Bacillus* spores were measured to provide insight into the spore germination process [96,106]. Ca-DPA is important in spore resistance to environmental stresses and in spore stability, and Ca-DPA levels in spore populations can vary with spore species/strains, as well as with sporulation conditions. Figure 17 shows some representative Raman spectra of single dormant and germinated *Bacillus* spores, as well as the spectra of Ca-DPA, and DPA [96].

## 4. Conclusions

The combination of optical trapping with Raman spectroscopy has proven itself to be a versatile and powerful tool in the study of biological particles. LTRS enables the measurement of Raman spectra from individual particles for applications ranging from particle detection and identification to longitudinal studies of the response of a biological particle such as a cell to environmental changes. Optical tweezers enable the localized measurement of Raman spectra from varying positions within a cell, as well as providing multi-modality cell characterization by using, for example, the optical tweezers to measure the mechanical properties of a particle while the Raman spectrum provides information about the chemical makeup of the particle. The ability to study individual particles, as opposed to collecting the combined Raman spectrum from a population of cells, provides additional information about the heterogeneity of the cells and the variation in the cell-to-cell response to environmental changes. This unique functionality has enabled researchers to identify the fast release of calcium dipicolinate in yeast cells, as well as to identify the fraction of cancer cells which respond to chemotherapy.

Although optical trapping holds particles in place long enough to make Raman measurements possible, the long exposure time still imposes a limitation on the throughput of LTRS systems. This limited throughput could be particularly challenging in on-line particle characterization techniques which use LTRS to identify airborne biological particles or cells in a microfluidic environment. As a result, a promising new area of research combines advances in stimulated Raman scattering or coherent anti-stokes Raman measurements with optical trapping. 
